# Molecular Mechanisms of NAFLD in Metabolic Syndrome

**DOI:** 10.1155/2015/621080

**Published:** 2015-05-20

**Authors:** Maria João Martins, António Ascensão, José Magalhães, Maria Carmen Collado, Piero Portincasa

**Affiliations:** ^1^Department of Biochemistry, Faculty of Medicine and Institute for Research and Innovation in Health, University of Porto, Porto, Portugal; ^2^Research Centre in Physical Activity, Health and Leisure (CIAFEL), Faculty of Sport, University of Porto, Porto, Portugal; ^3^Department of Biotechnology, Institute of Agrochemistry and Food Technology, Spanish National Research Council (IATA-CSIC), Valencia, Spain; ^4^Department of Biomedical Sciences and Human Oncology, Clinica Medica “A. Murri”, University of Bari Medical School, Bari, Italy

Metabolic syndrome (MetSyn) and nonalcoholic fatty liver disease (NAFLD) are increasing worldwide, as often mentioned in this special issue. Lifestyles (with diet being one of the relevant factors) play an important role as preventive determinants of MetSyn and NAFLD, conversely being potential inducers of MetSyn and NAFLD. Liver steatosis has been considered not only the hepatic MetSyn manifestation but also one of the earliest MetSyn signs. Liver steatosis represents the initial step of the whole spectrum of NAFLD.

Review and experimental articles including both basic and clinical data, pursuing the cross-talk between obesity/insulin resistance/MetSyn features and diet, lipid metabolism, redox state, oxidative and endoplasmic reticulum stress, mitochondrial dysfunction, inflammatory processes and mediators, glucocorticoid excess, developmental programming, and gut microbiota in NAFLD, are included in this special issue. Possible strategies for intervention against NAFLD are also presented and discussed.

There is growing evidence that the transcription factor nuclear factor erythroid-2 related factor 2 (Nrf2), a key regulator of cellular antioxidant responses, is also implicated in the regulation of hepatic lipid metabolism. The topic is addressed in the article of S. S. Chambel et al. N. Duarte et al. comprehensively reviewed the role of Kupffer cells on inflammation under conditions of hepatic fat deposition. E. Lau et al. analyzed in detail the recent research on the contribution of gut microbiota to NAFLD. A broad and extensive review on the growing evidences regarding the developmental programming of NAFLD by both maternal obesity and undernutrition is presented (M. Li et al.).

E. Maslak et al. showed that rats fed with a MetSyn-inducing diet supplemented with conjugated linoleic acid isomers (c9t11 and t10c12) had (globally) beneficial effects on the hepatic lipid content and glycogen accumulation as well as on the circulating lipid profile; the isomers also modulated the fatty acid composition and decreased lipogenic enzymes mRNA expression in the liver. A. Cordeiro et al. found that, in class III obesity, triglycerides, HDL-cholesterol and insulin levels (as well as insulin resistance index and waist circumference values), independently considered MetSyn features, linked to NAFLD, diagnosed by liver biopsy.

The analyzed topics included in the present special issue allow a better mechanistic understanding of NAFLD pathology in the context of the MetSyn and further contribute to the possible implementation of important countermeasures, either preventive or therapeutic, against the disease. Therefore, we hope that this special issue will be of interest to a wide audience in basic and applied biomedical areas, especially those that embrace with excitement the search for new hypothesis and approaching challenges regarding NAFLD pathophysiology in the context of the MetSyn.

*Maria João Martins* *António Ascensão* *José Magalhães* *Maria Carmen Collado* *Piero Portincasa*

